# Increased 1-deoxysphingolipids caused by an altered plasma alanine to serine ratio are associated with metabolic dysfunction-associated steatotic liver disease (MASLD)

**DOI:** 10.1007/s11306-025-02359-4

**Published:** 2025-11-02

**Authors:** F. Wipfli, M. A. Lone, A. von Eckardstein, A. Verrijken, S. Francque, J. Weyler, L. Van Gaal, B. Staels, T. Hornemann

**Affiliations:** 1 Institute of Clinical Chemistry, University Hospital Zurich and University of Zurich, 8091 Zurich, Switzerland; 2 Department of Gastroenterology Hepatology, Antwerp University Hospital, Drie Eikenstraat 655, B-2650 Edegem, Belgium; 3 Department of Endocrinology, Diabetology and Metabolism, Antwerp University Hospital, Drie Eikenstraat 655, B-2650 Edegem, Belgium; 4 InflaMed Centre of Excellence, Laboratory for Experimental Medicine and Paediatrics, Translational Sciences in Inflammation and Immunology, Faculty of Medicine and Health Sciences, University of Antwerp, Universiteitsplein 1, B-2610 Wilrijk, Belgium; 5 University of Lille, Inserm, CHU Lille, Institut Pasteur de Lille, U1011- EGID, Lille, France

## Abstract

Metabolic Dysfunction-Associated Steatotic Liver Disease (MASLD) (formerly Non-Alcoholic Fatty Liver Disease, NAFLD) encompasses a spectrum of metabolic disorders ranging from isolated steatosis to Metabolic Dysfunction-Associated Steatohepatitis (MASH), potentially progressing to cirrhosis and hepatocellular carcinoma (HCC). In this study, we investigated metabolic changes in MASLD by analysing plasma lipidomics and metabolomics profiles from 315 biopsy-characterized patients. We observed significant alterations in alanine/serine (ala/ser) ratio, 1-deoxysphingolipids, alanine aminotransferase (ALT), and waist/hip ratio (whr) between patients with and without MASH. These findings highlight a close interplay between amino acid metabolism and sphingolipid biosynthesis in MASLD progression. The shift in ala/ser ratio particularly distinguished non-MASH from borderline MASH patients, suggesting that early metabolic disruptions precede overt liver damage. Additionally, elevated branched-chain and aromatic amino acids correlated with steatosis severity, reinforcing the central role of amino acid dysregulation in MASLD. While a simple model combining ALT, ala/ser ratio, and whr showed some potential to support risk stratification, the primary significance of our findings lies in the mechanistic insights into metabolic dysfunction. In conclusion, this study emphasizes the importance of metabolic network alterations in MASLD and points toward future opportunities for both mechanistic research and the development of supportive diagnostic strategies.

**Graphical abstract:**

**Figure:**
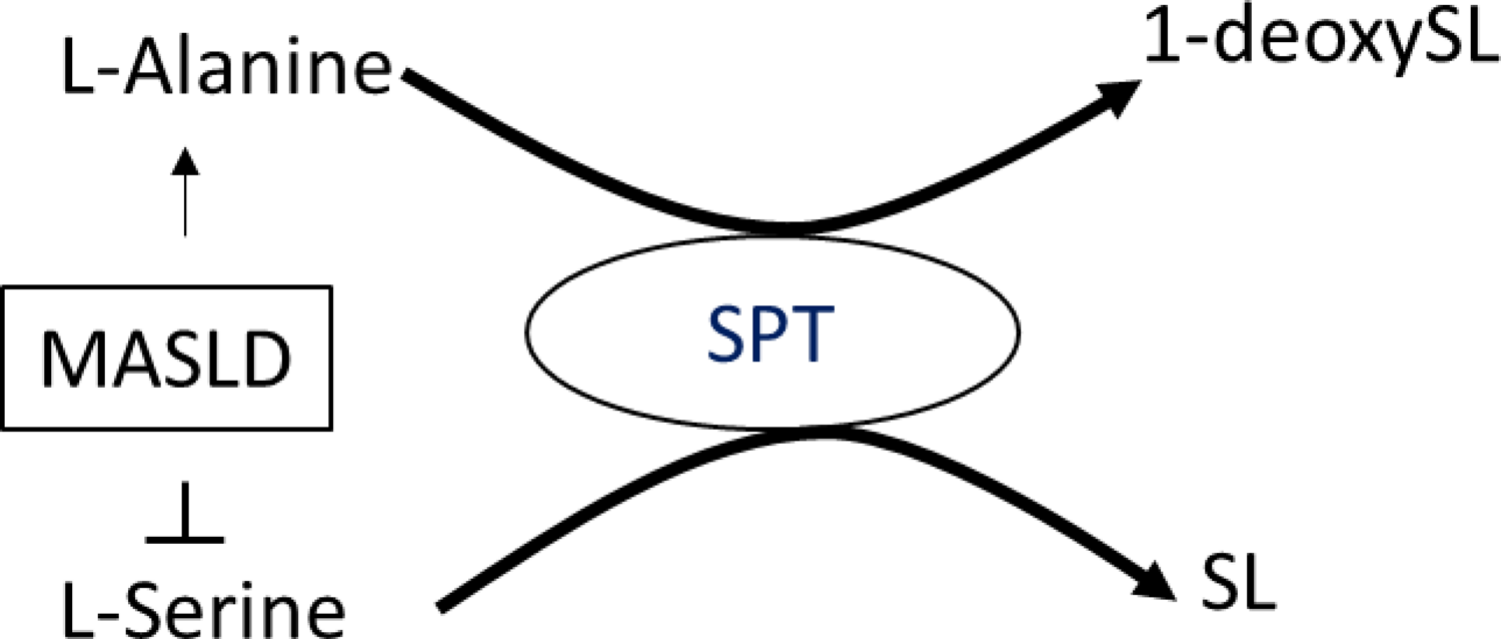
MASLD is associated with an increased ala/ser ratio which results in a metabolic shift from canonical SL to toxic 1-deoxySL

**Supplementary Information:**

The online version contains supplementary material available at 10.1007/s11306-025-02359-4.

## Introduction

Non-Alcoholic Fatty Liver Disease (NAFLD), recently renamed Metabolic Dysfunction-Associated Steatotic Liver Disease (MASLD), is characterized by abnormal lipid synthesis and accumulation in the liver, independent of significant alcohol consumption or other secondary causes. MASLD spans a spectrum from isolated steatosis (MASL) to Metabolic Dysfunction-Associated Steatohepatitis (MASH) and fibrosis, potentially progressing to cirrhosis and hepatocellular carcinoma (HCC) (Chalasani et al., 2018; Li et al., 2014; Eslam et al., 2018). It is now the leading cause of chronic liver disease in the Western world, with the highest prevalence in the Middle East and South America (Younossi et al., 2016; Alkhouri & Feldstein, 2016), and a global prevalence projected to exceed 30% by 2030 (Younossi et al., 2016; Estes et al., 2018).

The pathogenesis of MASLD remains incompletely understood. The original “two-hit” hypothesis has evolved into a “multiple-hit” model, involving increased adipose lipolysis and de novo lipogenesis, leading to excess free fatty acids (FFAs), free cholesterol, triglycerides (TGs), and other lipids in the liver. FFA accumulation drives mitochondrial dysfunction, reactive oxygen species (ROS) production, and hepatic inflammation (Buzzetti et al., 2016). Besides genetic predisposition (Sookoian & Pirola, 2017), MASLD is strongly associated with components of the metabolic syndrome (MetS) such as obesity, insulin resistance (IR), diabetes mellitus type 2 (T2DM), hypertension, and hypertriglyceridemia (Leite et al., 2009; Caballería et al., 2010). Consequently, MASLD is often regarded as the hepatic manifestation of MetS (Caballería et al., 2010; Marchesini et al., 2005; Loria et al., 2005).

Histologically, MASL is defined by steatosis in ≥ 5% of hepatocytes, while MASH additionally features lobular inflammation and hepatocellular ballooning (Chalasani et al., 2018). MASH can be identified in approximately 30% of patients diagnosed with MASLD by Ultrasound (Williams et al., 2011), and it carries an increased risk for cirrhosis, HCC, and cardiovascular events (Caligiuri et al., 2016; Lindenmeyer & McCullough, 2018). Although fibrosis is not a diagnostic criterion for MASH (Brunt & Tiniakos, 2010), it significantly worsens prognosis (Chalasani et al., 2018).

Given the high prevalence of MASLD, there is a critical need to monitor disease progression, particularly in high-risk groups. Early identification hinges on detecting specific biomarkers. Currently, liver biopsy remains the gold standard for diagnosing and staging MASLD (Cobbina & Akhlaghi, 2017), but its invasiveness, cost, and need for expert interpretation (Chalasani et al., 2018; Cobbina & Akhlaghi, 2017) highlight the urgent need for reliable, non-invasive alternatives.

Traditional serum markers of liver injury, such as alanine aminotransferase (ALT), aspartate aminotransferase (AST), and gamma-glutamyl transferase (GGT), show limited sensitivity and specificity in MASLD (Leite et al., 2009). Over half of MASLD patients have normal GGT levels (Sanyal et al., 2015), and disease can be present despite normal ALT values (Mofrad et al., 2003). Elevated ALT also does not reliably indicate severity of MASH or fibrosis (Maximos et al., 2015; Fracanzani et al., 2008).

Recent efforts have focused on identifying circulating proteins—hormones, cytokines, adipokines, and carrier proteins—as biomarkers for MASLD and MASH (Wong et al., 2018). However, these candidates face challenges related to clinical implementation and limited stage specificity. Emerging OMICS technologies, particularly lipidomics and metabolomics, may overcome these limitations and improve early detection of disease progression.

Given the overlapping pathophysiology of MASLD, MetS, and T2DM (Mikolasevic et al., 2016), biomarkers identified in these conditions may also reflect MASH risk. Among lipid classes, sphingolipids (SL), particularly ceramides, have been implicated in insulin resistance, T2DM, and MASLD (Chaurasia et al., 2019). SLs play critical roles in cellular processes (Simons & Ikonen, 1997), and elevated levels in liver and plasma are associated with liver dysfunction, IR, and steatosis (Ichi et al., 2007; Xia et al., 2014). Circulating ceramides may directly impair insulin signalling (Holland et al., 2007), and enzymes regulating SL metabolism are considered therapeutic targets (Hammerschmidt et al., 2019; Raichur et al., 2019; Turpin-Nolan et al., 2019). Recent OMICS studies have also linked an atypical class of sphingolipids, 1-deoxysphingolipids (1-deoxySLs), to MASLD, MetS, and T2DM (Gorden et al., 2015; Weyler et al., 2020; Othman et al., 2011; Bertea et al., 2010).

1-DeoxySLs are formed through an alternate reaction of de novo sphingolipid synthesis when serine availability is reduced, leading serine-palmitoyltransferase to utilize alanine (Zitomer et al., 2009; Lone et al., 2019). These aberrant products lack the C1-hydroxyl group essential for canonical sphingolipid function and degradation, contributing to toxicity (Lone et al., 2019).

Building on these findings, we aimed to investigate whether canonical and non-canonical sphingolipids, together with metabolic parameters, could reflect histological stages of MASLD, offering new insights into disease mechanisms and potential non-invasive diagnostic strategies.

## Patients and methods

### Study

Patients attending the obesity clinic of the Antwerp University Hospital were consecutively included in the study upon informed consent. A metabolic work-up, laboratory analyses and a liver specific-program (to exclude other liver diseases than MASLD) were performed.

Patients presenting to the obesity clinic underwent a detailed metabolic and liver assessment, including an extensive panel of coagulation factors. If MASLD was suspected, a liver biopsy was proposed. A series of 273 consecutive patients (65% female) with a liver biopsy were included (age, 44 ± 0.76 years; body mass index: 39.6 ± 0.40 kg/m2). Patients had to be 18 years or older, had no significant alcohol consumption and no history of diabetes (as medical treatment of the latter may substantially influence liver histology; *de novo* diagnosis of T2DM was, however, allowed). For more information, refer to a previous description of the patient cohort (Verrijken et al., 2014).

Pathological features like steatosis grade, ballooning, lobular inflammation, and fibrosis stage were scored according to the NASH Clinical Research Network Scoring System (NASH-CRN) (Kleiner et al., 2005). For the classification of patients based on the severity of MASLD we considered two different approaches. On one hand, we distinguished between No-MASH and MASH (Brunt et al., 1999), with MASH being defined as the co-existence of steatosis, ballooning, and lobular inflammation (hence a score of 1 or more for each). On the other hand, we classified the patients more granularly according to the NAFLD/MASLD activity score (NAS/MAS), which is defined by the sum of the scores for steatosis, ballooning, and lobular inflammation. The criteria for the classification are as follows: MAS < 3 (No-MASH); MAS 3–4 (borderline-MASH); and MAS ≥ 5 (definite MASH) (Kleiner et al., 2005); Brunt et al., 2011).

### Sphingoid base analysis

Sphingoid bases were extracted from fasting plasma samples. Fifty microliters of plasma were filled up with phosphate-buffered saline to a volume of 100 µL. After mixing by vortex at room temperature, the lipids were extracted in 500 µL extraction buffer (499.8 µL methanol + 0.2 µL D7-labelled C18 sphingosine). After another short mixing, the lipids were extracted under constant agitation (1400 rpm) for 1 h at 37 °C. Subsequently samples were centrifuged (16100 rcf, 5 min) and 500 µL of the supernatant was transferred to a new tube. For acid and base hydrolysis, 75 µL of HCl (conc. 32%) was first added and mixed, and the samples were then stored at 65 °C for 12–15 h. After that, samples were neutralised by adding 100 µL of KOH (10 M). Lipid extraction was performed in the following steps: First, 125 µL of chloroform (CHCl_3_) was added and mixed. Then another 500 µL of CHCl_3_ was added and mixed again. Finally, 100 µL of 2 N ammonia and 0.5 mL of alkaline water were added and samples were centrifuged (16000 rcf) for 5 min. The upper phase was removed, and the lower phase was washed twice again with 1 mL alkaline water. The lipids were then dried under N_2_. Dried lipids were resuspended in 70 µL of reconstitution buffer (70% methanol, 10 mM ammonium acetate) and subjected to LC-MRM-MS analysis. The separation of sphingoid bases has been described previously (Lone et al., 2020). Quantification was performed using MultiQuant (2.1) software (SCIEX) and normalized to the internal standard D7-C18 sphingosine.

### Sphingoid base 1-phosphate analysis

Fifty microliter of fasting plasma was filled up with ddH_2_O to a volume of 100 µL. The lipids were then extracted in 400 µL extraction solvent (50 nM D7-S1P in methanol), mixed and extracted under constant agitation (1400 rpm) for 1 h at 37 °C. After that, samples were centrifuged at 14,000 rcf for 10 min. Subsequently, the supernatant was removed, transferred to a new tube, and dried under N_2_. The dried samples were re-dissolved in 50 µL reconstitution solvent (95% methanol, 10 mM ammonium acetate) and shaken for 30 min (950 rpm, 25 °C). After centrifugation for 15 min at 14,000 rcf the supernatants were transferred to a new tube and analysed by LC-MRM-MS. Sphingoid base 1-phosphates (S1P) were separated via a reverse-phase C18 column (Luna Omega Polar C18, 150 × 2.1 mm) connected to a QTRAP 6500 + LC-MS/MS-MS System (Sciex), operated in multiple reaction monitoring mass spectrometry mode. Sample ionisation was achieved via electrospray ionisation in alternating positive and negative ion mode. For chromatography, a binary solvent system consisting of solvent A (5 mM ammonium formate in H_2_O/methanol/formic acid (20/80/0.5, v/v/v)) and solvent B (5 mM ammonium formate in methanol/acetonitrile/formic acid (60/40/0.5, v/v/v)) was used. The flow rate was maintained at 0.3 mL/min throughout. The column was equilibrated with 100% solvent A, and a linear gradient to 100% B was run over 4 min and washed with 100% B for 4 min before equilibration in 100% A for 6 min. The acquired data were integrated and analysed using the open source software tool Skyline (DOI: 10.1021/acs.jproteome.9b00640). For normalisation and quantification, the internal standard D7-S1P was added to the samples.

### Amino acid analysis

To 10 µL of serum, 1 nmol of stable isotope labelled amino acids were added (Cambridge Isotope Laboratories). Proteins were precipitated by adding 15 volumes ice cold methanol at −20 °C. After incubation for 30 min at −20 °C, samples were centrifuged for 10 min at 4 °C (14000 rcf). The supernatant was dried under N_2_ and the amino acids were re-constituted in 100 µL 0.1% acetic acid for 1 h at 24 °C shaking at 950 rpm. After centrifugation for 5 min at 16100 rcf the supernatant was analysed by LC-MRM-MS.

Amino acids were separated via a reverse-phase C18 column (EC 250/2 NUCLEOSIL 100-3 C18HD, L = 250 mm, ID: 2 mm; Macherey-Nagel). 5 µL per sample were subjected to liquid chromatography coupled multiple reaction monitoring mass spectrometry using a QTRAP 6500 + LC-MS/MS-MS System (Sciex). The amino acids were chromatographed isocratically with 0.1% formic acid in water for five minutes, followed by a linear gradient to 50% solvent B (acetonitrile) over two minutes. Then, the column was washed with 80% solvent B prior equilibration with 100% solvent A. The flow rate was held constant at 0.2 mL/min. Sample ionization was achieved via electrospray ionization in positive ion mode. Quantification was performed using MultiQuant (2.1) software (SCIEX) and normalised to internal standards.

### Statistical analysis

Statistical tests were performed using IBM SPSS version 27. Mean values were compared using either independent t-test or one-way ANOVA, followed by Bonferroni correction. In case of unequal variances, comparisons were conducted by Welch test followed by Bonferroni correction and Welch-ANOVA with Games-Howell correction. Where data were skewed, log or square root transformation was used in statistical analysis. To investigate the association of anthropometrical features, metabolic parameters, values of liver tests, sphingoid bases and amino acids within different stages of MASLD, we compared the following groups: No-MASH versus MASH dichotomously; No-MASH versus borderline-MASH versus definite MASH according to MAS.

The regression coefficient β of multivariate binary regression models was calculated using IBM SPSS. Z-Scores and prediction function was calculated in Excel. Receiver operating characteristic (ROC) curve analysis was performed using MetaboAnalyst 5.0.

## Results

### Cohort characteristics

This study is based on the HEPADIP cohort that was initiated by the Antwerp University Hospital (reference 6/25/125, Belgian registration number B30020071389) and established as part of the FP6-Lifescihealth program (https://cordis.europa.eu/project/id/18734/de).

It consists of clinically very well characterised individuals and comprises > 180 baseline parameters including liver histology. A total of 315 individuals were included in this analysis (217 women and 98 men). Mean age was 43.7 (±12.5) years, and mean body mass index (BMI) was 39.3 (±5.9) kg/m^2^. Based on the definition of MASH, 45.1% (*n* = 142) patients were diagnosed with no-MASH and 54.9% (*n* = 173) with MASH. Based on the MAS score, 39% were No-MASH (*n* = 123), 26.7% borderline MASH (*n* = 84) and 34.3% definite MASH (*n* = 108).

### Differences between no-MASH and MASH

Anthropometric features, clinical laboratory values, metabolic markers, the sphingoid base and amino acid profiles were compared between the No-MASH and MASH dichotomous groups (Table 1). The most significant differences between the groups were seen for ALT (*p* = 2.04E-10), followed by the alanine/serine (ala/ser) ratio (*p* = 6.92E-09) and the waist/hip-ratio (whr) (*p* = 3.46E-08). Although less pronounced, significant differences were also seen for AST (*p* = 6.83E-07) and GGT (*p* = 2.27E-02). No significant differences in sphingoid base levels including sphingoid-1-phohsphates were observed except for 1-deoxySO that was significantly increased in MASH (*p* = 3.27E-03), confirming earlier findings (Weyler et al., 2020).

For the amino acids, we found that alanine (*p* = 2.22E-05), tyrosine (*p* = 3.02E-05), phenylalanine (*p* = 5.70E-03) and the branched chain AA (BCAA) valine (*p* = 6.31E-04), leucine (*p* = 1.25E-03) and isoleucine (*p* = 1.67E-03) were significantly higher in MASH. In addition, variables typically associated with MetS such as plasma triglycerides (TG) (*p* = 8.73E-03), HbA1c (*p* = 2.41E-02), fasting glucose *(p* = 2.91E-02) and fasting insulin (*p* = 1.28E-02) were significantly elevated in the MASH group. However, no differences were seen for total cholesterol (total-C), high-density lipoprotein cholesterol (HDL-C), and low-density lipoprotein cholesterol (LDL-C). The use of lipid lowering medication did not significantly differ between the two groups.

As about 70% of the patients were female, we also compared the groups separated by gender (supplementary Table S1and S2).

**Table 1:** Clinical and laboratory data according to the definition of MASH

	Total *N* = 315 (100%)		No-MASH *N* = 142 (45.1%)		MASH *N* = 173 (54.9%)		*P* value	Adjusted *p* value	Significance level
Age (years)	43.7	(±12.5)	41.8	(±12.1)	45.2	(±12.6)	1.63E-02	0.978^*†*^	NS
**Gender: men**	**98**	**(31.1%)**	**26**	**(18.3%)**	**72**	**(41.6%)**	**8.74E-06** ^***#***^		****
Weight (kg)	111.9	(±19.8)	109.5	(±17.9)	113.9	(±21.1)	6.66E-02	1.00^*†*^	NS
BMI (kg/m^2^)	39.3	(±5.9)	39.2	(±6.0)	39.4	(±5.8)	0.706	1.00^*†*^	NS
**Waist (cm)**	**118.3**	**(±13.1)**	**115.2**	**(±13.2)**	**120.8**	**(±12.4)**	**7.53E-05**	**4.52E-03** ^***†***^	**
Hip (cm)	122.6	(±10.4)	124.4	(±10.1)	121.1	(±10.6)	4.48E-03	0.269^*†*^	NS
**Waist/Hip-Ratio**	**1.0**	**(±0.1)**	**0.9**	**(±0.1)**	**1.0**	**(±0.1)**	**5.77E-10**	**3.46E-08** ^***†***^	****
Total-C (mg/dL)	203.3	(±40.1)	202.2	(±42.2)	204.1	(±38.3)	0.621	1.00^*†*^	NS
HDL-C (mg/dL)	49.8	(±14.2)	52.5	(±14.6)	47.5	(±13.4)	9.01E-04	5.40E-02^*†*^	NS
LDL-C (mg/dL)	123.7	(±36.1)	122.6	(±37.6)	124.6	(±34.9)	0.574	1.00^*†*^	NS
**TG (mg/dL)**	**146.9**	**(±67.4)**	**130.9**	**(±58.0)**	**160.1**	**(±71.8)**	**1.45E-04**	**8.73E-03** ^***†***^	**
**HbA1c (%)**	**5.6**	**(±0.5)**	**5.5**	**(±0.3)**	**5.7**	**(±0.6)**	**4.02E-04**	**2.41E-02** ^***‡***^	*
**Glucose (mg/dL)**	**85.6**	**(±21.4)**	**81.8**	**(±9.6)**	**88.7**	**(±27.1)**	**4.85E-04**	**2.91E-02** ^***‡***^	*
**Insulin (µU/dL)**	**17.5**	**(±11.5)**	**15.3**	**(±10.8)**	**19.3**	**(±11.8)**	**2.13E-04**	**1.28E-02** ^***†***^	*
Medication for diabetes	0	(0%)	0	(0%)	0	(0%)			NS
Lipid lowering medication	34	(10.8%)	11	(7.7%)	23	(13.3%)	0.114^*#*^		NS
**AST (U/L)**	**30.8**	**(±17.0)**	**25.3**	**(±7.9)**	**35.4**	**(±20.6)**	**1.14E-08**	**6.83E-07** ^***‡***^	****
**ALT (U/L)**	**44.4**	**(±26.6)**	**34.6**	**(±15.7)**	**52.4**	**(±30.8)**	**3.41E-12**	**2.04E-10** ^***‡***^	****
**GGT (U/L)**	**40.7**	**(±28.6)**	**36.1**	**(±26.5)**	**44.4**	**(±29.8)**	**3.79E-04**	**2.27E-02** ^***†***^	*
Total bilirubin (mg/dL)	0.55	(±0.25)	0.50	(±0.21)	0.59	(±0.27)	1.18E-03	7.10E-02^*†*^	NS
CRP (mg/dL)	0.8	(±0.9)	0.9	(±1.0)	0.7	(±0.8)	9.27E-02	1.00^*†*^	NS
CDT	1.89	(±0.39)	1.91	(±0.44)	1.88	(±0.34)	0.513	1.00^*†*^	NS
*Sphingoid bases* (µM)									
C16SO	17.013	(±5.223)	17.319	(±5.022)	16.762	(±5.384)	0.285	1.00^*†*^	NS
C16SA	0.665	(±0.279)	0.620	(±0.226)	0.701	(±0.313)	1.19E-02	0.712^*†*^	NS
C17SO	7.695	(±1.947)	7.817	(±1.890)	7.594	(±1.993)	0.250	1.00^*†*^	NS
C17SA	0.102	(±0.040)	0.095	(±0.034)	0.108	(±0.043)	3.56E-03	0.214^*†*^	NS
C18SAdiene	34.487	(±8.222)	35.613	(±8.209)	33.562	(±8.140)	2.21E-02	1.00^*†*^	NS
C18SO	74.879	(±13.227	76.277	(±13.699)	73.730	(±12.751)	9.36E-02	1.00^*†*^	NS
C18SA	3.804	(±1.189)	3.689	(±1.147)	3.897	(±1.218)	0.127	1.00^*†*^	NS
C19SO	0.235	(±0.081)	0.228	(±0.080)	0.241	(±0.081)	0.129	1.00^*†*^	NS
C19SA	0.010	(±0.006)	0.009	(±0.006)	0.010	(±0.006)	0.423	1.00^*†*^	NS
MeC18SO	2.511	(±0.908)	2.492	(±0.856)	2.526	(±0.952)	0.869	1.00^*†*^	NS
MeC18SA	0.113	(±0.052)	0.104	(±0.047)	0.120	(±0.054)	6.85E-03	0.411^*†*^	NS
C18PhytoSO	0.866	(±0.331)	0.881	(±0.342)	0.853	(±0.321)	0.440	1.00^*†*^	NS
C20SO	0.185	(±0.064)	0.184	(±0.064)	0.186	(±0.065)	0.846	1.00^*†*^	NS
C20SA	0.022	(±0.014)	0.021	(±0.013)	0.023	(±0.015)	0.253	1.00^*†*^	NS
**1-deoxySO**	**0.236**	**(±0.134)**	**0.204**	**(±0.113)**	**0.262**	**(±0.144)**	**5.45E-05**	**3.27E-03** ^***†***^	**
1-deoxySA	0.135	(±0.069)	0.126	(±0.062)	0.143	(±0.074)	2.44E-02	1.00^*†*^	NS
C16SO-1-phosphate	0.033	(±0.017)	0.034	(±0.016)	0.033	(±0.018)	0.300	1.00^*†*^	NS
C17SO-1-phosphate	0.019	(±0.011)	0.019	(±0.009)	0.018	(±0.012)	0.408	1.00^*†*^	NS
C18SAdiene-1-phosphate	0.382	(±0.158)	0.386	(±0.137)	0.378	(±0.174)	0.468	1.00^*†*^	NS
C18SO-1-phosphate	0.580	(±0.127)	0.592	(±0.129)	0.570	(±0.125)	0.133	1.00^*†*^	NS
C18SA-1-phosphate	0.115	(±0.045)	0.115	(±0.040)	0.114	(±0.048)	0.719	1.00^*†*^	NS
MeC18SO-1-phosphate	0.010	(±0.006)	0.010	(±0.006)	0.010	(±0.007)	0.827	1.00^*†*^	NS
C19SO-1-phosphate	0.004	(±0.003)	0.003	(±0.003)	0.004	(±0.003)	0.207	1.00^*†*^	NS
*Amino Acids (mM)*									
Glycine	0.079	(±0.027)	0.082	(±0.034)	0.076	(±0.020)	0.231	1.00^*‡*^	NS
**Alanine**	**0.148**	**(±0.034)**	**0.137**	**(±0.032)**	**0.156**	**(±0.034)**	**3.71E-07**	**2.22E-05** ^***†***^	****
Serine	0.036	(±0.008)	0.037	(±0.008)	0.035	(±0.007)	7.24E-02	1.00^*†*^	NS
**Alanine/Serine-ratio**	**4.234**	**(±1.060)**	**3.8**	**(±0.9)**	**4.6**	**(±1.1)**	**1.15E-10**	**6.92E-09** ^***†***^	****
Proline	0.084	(±0.024)	0.079	(±0.024)	0.087	(±0.024)	**2.13E-03**	0.128^*†*^	NS
**Valine**	**0.122**	**(±0.026)**	**0.115**	**(±0.024)**	**0.128**	**(±0.026)**	**1.05E-05**	**6.31E-04** ^***†***^	***
Threonine	0.058	(±0.014)	0.059	(±0.014)	0.058	(±0.014)	0.597	1.00^*†*^	NS
**Leucine**	**0.029**	**(±0.007)**	**0.027**	**(±0.006)**	**0.031**	**(±0.007)**	**2.08E-05**	**1.25E-03** ^***†***^	**
**Isoleucine**	**0.071**	**(±0.016)**	**0.067**	**(±0.014)**	**0.074**	**(±0.016)**	**2.79E-05**	**1.67E-03** ^***†***^	**
Asparagine	0.010	(±0.002)	0.009	(±0.002)	0.010	(±0.002)	0.154	1.00^*†*^	NS
Lysine	0.100	(±0.021)	0.098	(±0.022)	0.102	(±0.019)	0.103	1.00^*†*^	NS
Glutamine	0.090	(±0.027)	0.086	(±0.024)	0.093	(±0.029)	1.64E-02	0.986^*†*^	NS
Methionine	0.011	(±0.003)	0.010	(±0.003)	0.011	(±0.003)	1.50E-02	0.900^*†*^	NS
Histidine	0.046	(±0.009)	0.046	(±0.010)	0.046	(±0.008)	0.540	1.00^*†*^	NS
Arginine	0.032	(±0.016)	0.033	(±0.018)	0.031	(±0.014)	0.210	1.00^*†*^	NS
**Tyrosine**	**0.037**	**(±0.010)**	**0.034**	**(±0.009)**	**0.040**	**(±0.010)**	**5.03E-07**	**3.02E-05** ^***†***^	****
Cysteine	0.004	(±0.002)	0.004	(±0.003)	0.004	(±0.002)	0.416	1.00^*†*^	NS
**Phenylalanine**	**0.050**	**(±0.007)**	**0.048**	**(±0.008)**	**0.051**	**(±0.007)**	**9.50E-05**	**5.70E-03** ^***†***^	**

No histological evidence of Metabolic Dysfunction-Associated Steatohepatitis (No-MASH), Metabolic Dysfunction-Associated Steatohepatitis (MASH) according to Brunt, body mass index (BMI), total cholesterol (Total-C), high-density lipoprotein cholesterol (HDL-C), low-density lipoprotein cholesterol (LDL-C), triglycerides (TG), glycated haemoglobin A1 (HbA1c), aspartate aminotransferase (AST), alanine aminotransferase (ALT), gamma-glutamyltransferase (GGT), C-reactive protein (CRP), carbohydrate-deficient transferrin (CDT), C16 sphingosine (C16SO), C16 sphinganine (C16SA), C17 sphingosine (C17SO), C17 sphinganine (C17SA), C18 sphingadiene (C18SAdiene), C18 sphingosine (C18SO), C18 sphinganine (C18SA), C19 sphingosine (C19SO), C19 sphinganine (C19SA), omega-3-methylated C18 sphingosine (meC18SO), omega-3-methylated C18 sphinganine (meC18SA), C18 phyto-sphingosine (C18PhytoSO), C20 sphingosine (C20SO), C20 sphinganine (C20SA), 1-deoxysphingosine (1-deoxySO), 1-deoxysphinganine (1-deoxySA). Data are expressed as mean ±SD or percentage. p values: † Unpaired t test followed by Bonferroni multiple correction, ‡ Welch test followed by Bonferroni multiple correction, #Pearson Chi-square. Significant differences are shown in bold. *P < 0.05, **P < 0.01, ***P < 0.001, ****P < 0.0001.

### Binary logistic regression analysis of MASH versus no-MASH

**Fig. 1:**
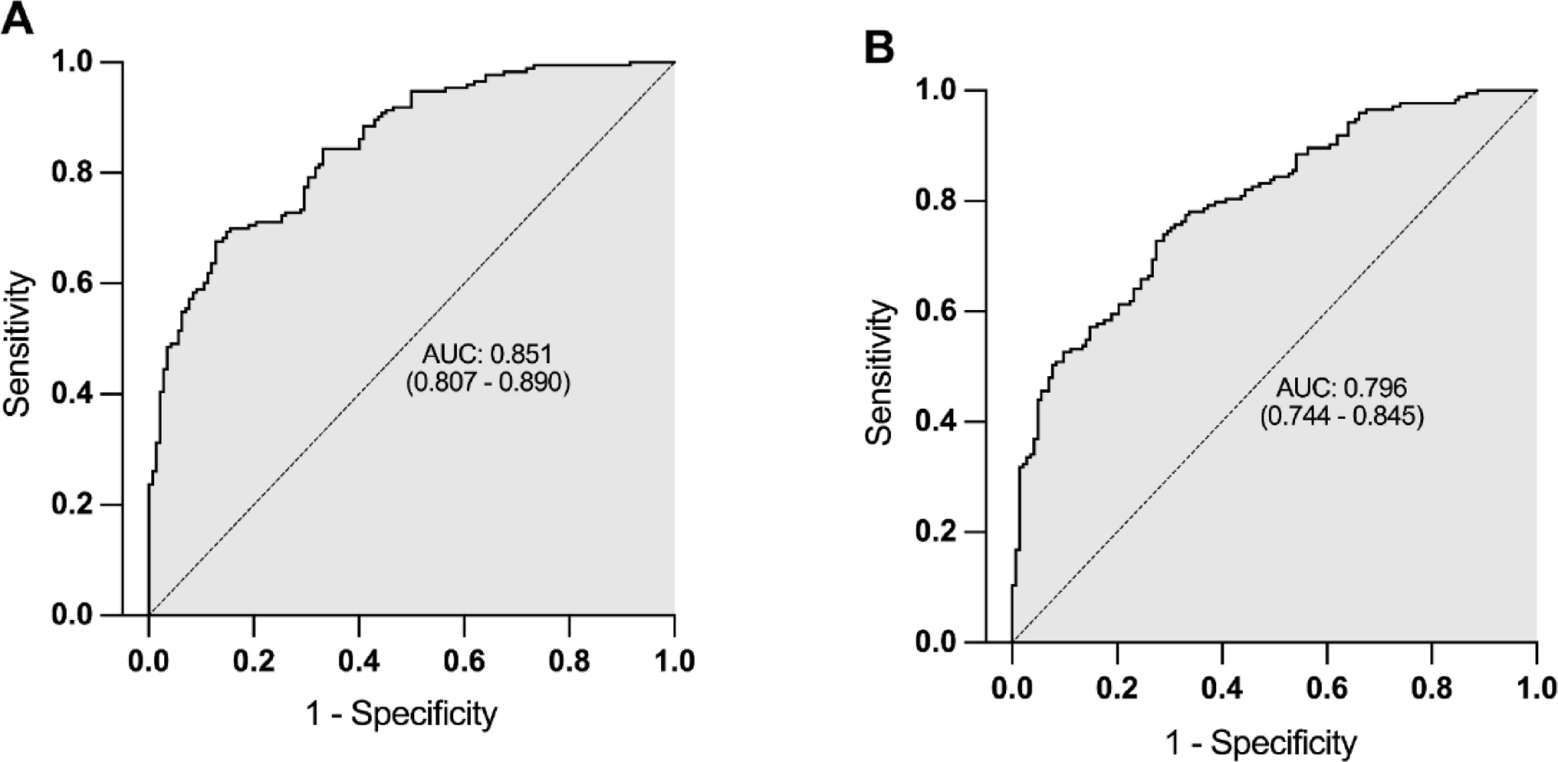
ROC curves for No-MASH versus MASH of two multivariate binary regression models. **A** The model contains whr, ALT, GGT, doxSO, doxSA, C17SA, MeC18SO, alanine, serine, asparagine and histidine as independent variables and shows an AUC of 0.851 (*p* = 8.18E-29). **B** Potential biomarker model showing whr, ALT, alanine and serine as predictors of MASH with AUC of 0.796 (*p* = 6.79E-21)

We calculated different multivariate binary regression models using MASH versus No-MASH as dependent variable and optimised independent variables by stepwise conditional forwarding. The diagnostic potential of the combined markers was analysed in an receiver operator curve (ROC) curve analysis (Fig. 1A, B). Optimal cut-off was set on closest point to top-left corner.

The most complex model (model 1) including whr, ALT, GGT, 1-deoxySO, 1-deoxySA, C17SA, MeC18SO, alanine, serine, asparagine and histidine as independent variables (Fig. 1A) showed with 0.851 (*p* = 8.18E-29) the highest area under the curve (AUC) resulting in a sensitivity of 70.0% and a specificity of 84.5% for the distinction between MASH and no-MASH.

The model with the highest AUC in relation to the number of variables is shown in Fig. 1B (model 2). The signature consists of whr, ALT, alanine and serine as independent variables and showed an AUC of 0.769 (*p* = 6.79E-21). Overall percentage of accuracy in classification was 72.7%, with a sensitivity of 72.8% and a specificity of 72.5%. All three variables included in the model contributed significantly to the prediction of MASH.

In contrast to the previous approach, the MAS score allows for a more graded classification of liver histology including the category borderline MASH. To verify the diagnostic potential of our biomarker signature, we applied the MAS score-based classification to Model 2 (whr, ALT, alanine and serine). Out of 84 patients with histologically boderline NASH, 34 (40.48%) were assigned to the no-MASH group and 50 (59.52%) assigned to the MASH group. Distinguishing between no-MASH and borderline MASH (excluding the group of definite MASH), Model 2 was able to allocate 69.57% correctly. Although it correctly classified 116 out of 123 No-MASH patients (94.31%), only 28 out of 84 borderline-Patients (33.33%) were classified correctly.

## Discussion

Liver biopsy remains the gold standard for diagnosing MASH (Brunt et al., 1999), yet its invasive nature and limitations in routine diagnostics underline the ongoing need for non-invasive strategies to better understand and monitor disease progression. While several biomarker-based approaches have been proposed to address this gap, it is becoming increasingly evident that a deeper understanding of the underlying metabolic pathways may offer more meaningful clinical insight—particularly in the context of the recently redefined term MASLD.

In this study, we identified a strong association between MASLD and specific shifts in amino acid metabolism, most notably an elevated alanine to serine (ala/ser) ratio. This finding is of particular interest given its mechanistic connection to sphingolipid and particularly 1-deoxysphingolipid biosynthesis—an atypical pathway that is activated when serine becomes limiting and alanine is used as an alternative substrate by serine-palmitoyltransferase. The observed increase in 1-deoxySL levels in MASH patients, therefore, not only supports a metabolic imbalance but may also reflect a broader dysregulation of the alanine and serine metabolism influencing sphingolipid homeostasis in MASLD.

Previous studies have reported altered alanine levels in MASLD, although many lacked histological confirmation or did not measure serine. Hasegawa et al. (Hasegawa et al., 2020) found that alanine was the only amino acid significantly different between MASLD patients and healthy controls after adjusting for insulin resistance, but they did not explore this further. Pietzner et al. (Pietzner et al., 2018). also observed a positive link between liver fat (measured by MRI) and alanine levels, mainly influenced by insulin resistance. They also reported an inverse relationship between liver fat and serine levels, which was linked to triglycerides. However, neither of these studies used liver biopsy to confirm MASLD stages. Only Gaggini et al. (Gaggini et al., 2018). used histological criteria and reported higher alanine levels in MASLD patients, while L-serine levels remained unchanged.

Our findings expand on these by providing histology-based differentiation and linking the amino acid changes directly to sphingolipid metabolism. Other amino acids—including branched-chain and aromatic amino acids—were also elevated in MASH patients, consistent with earlier reports and reinforcing the idea that amino acid perturbations reflect systemic metabolic stress in MASLD.

Although we explored a logistic regression model combining simple clinical variables (whr, ALT, alanine, and serine), this was not intended as a definitive diagnostic tool. Rather, the aim was to demonstrate that even routine metabolic and anthropometric parameters reflect the metabolic shifts occurring with disease progression. These shifts, especially the altered alanine and serine levels, appear to precede more classical markers of liver damage and could serve as early indicators of metabolic dysfunction rather than standalone diagnostic biomarkers.

When applying the MAS score classification, the ala/ser ratio distinguished No-MASH from borderline MASH more clearly than ALT, suggesting that disruptions in amino acid metabolism may occur early in the disease process, before overt liver injury. This metabolic signal, coupled with the downstream impact on sphingolipid profiles, aligns with prior reports linking elevated alanine and reduced serine availability with hepatic steatosis, insulin resistance, and oxidative stress (Mardinoglu et al., 2017).

The connection between alanine, serine, and 1-deoxySLs is further supported by studies in glycogen storage diseases and metabolic syndrome, where similar patterns of metabolite shifts have been observed (Hornemann et al., 2018; Melis et al., 2015). In these contexts, altered substrate availability for sphingolipid synthesis led to increased production of cytotoxic 1-deoxySLs. Moreover, studies demonstrating the therapeutic potential of serine supplementation suggest that restoring amino acid balance could modify disease trajectory, further underlining the relevance of these pathways.

A strength of our study is the well-characterized cohort with biopsy-confirmed MASLD stages, enabling us to explore these metabolic alterations across a continuum of disease severity. However, limitations include the gender imbalance in the cohort and the absence of a non-obese control group, which may influence generalizability. Additionally, while the statistical model illustrates the relationship between variables, it was not cross-validated in an independent dataset, and we emphasize that the model serves an illustrative rather than predictive purpose.

In conclusion, our study highlights a potentially important metabolic axis, connecting amino acid imbalance to sphingolipid dysregulation, in the pathogenesis of MASLD. These findings emphasize the complexity of MASLD as a systemic metabolic disease and offer a framework for future studies focused on mechanistic validation and therapeutic targeting, rather than immediate clinical application as biomarkers.

## Supplementary Information

Below is the link to the electronic supplementary material.

Supplementary material 1 (DOCX 35.9 kb)

## Data Availability

Data are made available by the authors on request.
