# Supplementary material for: Increased 1-deoxysphingolipids caused by an altered plasma alanine to serine ratio are associated with metabolic dysfunction-associated steatotic liver disease (MASLD)
Source: Metabolomics. 2025 Nov 2;21(6):157. doi: 10.1007/s11306-025-02359-4 (PMC12580439; doi:10.1007/s11306-025-02359-4)
Supplement: Supplementary file 1 — Supplementary material 1 (DOCX 35.9 kb) [file 11306_2025_2359_MOESM1_ESM.docx]

# Supplementary Information

**Table S1** Characteristics in women without MASH versus MASH

| Women | Total  N=217 (100%) | | No-MASH  N=116 (53.5%) | | MASH  N=101 (46.5%) | | *p* value | Adjusted *p* value | Significance level |
| --- | --- | --- | --- | --- | --- | --- | --- | --- | --- |
| Age (years) | **43.3** | **(±12.4)** | **40.7** | **(±12.0)** | **46.3** | **(±12.2)** | **7.77E-04** | **4.66E-02***^†^* | ***** |
| Weight (kg) | 107.3 | (±16.8) | 107.2 | (±17.0) | 107.4 | (±16.7) | 0.915 | 1.00*^†^* | NS |
| BMI (kg/m^2^) | 39.6 | (±5.7) | 39.4 | (±6.1) | 39.8 | (±5.4) | 0.469 | 1.00*^†^* | NS |
| Waist (cm) | 115.8 | (±12.2) | 113.8 | (±12.9) | 118.2 | (±11.0) | **5.05E-03** | 0.303*^†^* | NS |
| Hip (cm) | 124.3 | (±10.1) | 125.7 | (±9.7) | 122.7 | (±10.4) | **2.24E-02** | 1.00*^†^* | NS |
| Waist/Hip-Ratio | **0.9** | **(±0.1)** | **0.9** | **(±0.1)** | **1.0** | **(±0.1)** | **2.77E-06** | **1.66E-04***^†^* | ******* |
| Total-C (mg/dl) | 206.7 | (±40.2) | 204.5 | (±40.9) | 209.2 | (±39.5) | 0.365 | 1.00*^†^* | NS |
| HDL-C (mg/dL) | 53.3 | (±14.8) | 55.1 | (±14.5) | 51.3 | (±15.0) | **3.27E-02** | 1.00*^†^* | NS |
| LDL-C (mg/dL) | 124.7 | (±36.4) | 123.2 | (±36.5) | 126.5 | (±36.4) | 0.487 | 1.00*^†^* | NS |
| TG (mg/dL) | **140.4** | **(±63.7)** | **126.0** | **(±52.7)** | **156.9** | **(±71.2)** | **4.09E-04** | **2.46E-02***^†^* | ***** |
| HbA1c (%) | 5.6 | (±0.4) | 5.5 | (±0.3) | 5.7 | (±0.5) | **1.86E-03** | 0.112*^‡^* | NS |
| Glucose (mg/dL) | 83.4 | (±13.1) | 80.8 | (±8.7) | 86.3 | (±16.3) | **2.91E-03** | 0.175*^‡^* | NS |
| Insulin (μU/dL) | 16.1 | (±9.4) | 14.4 | (±9.0) | 18.1 | (±9.5) | **1.84E-03** | 0.110*^†^* | NS |
| Lipid lowering medication | **19** | **(8.8%)** | **6** | **(5.2%)** | **13** | **(12.9%)** | **4.53E-02^#^** |  |  |
| AST (U/L) | **27.5** | **(±14.6)** | **24.2** | **(±7.5)** | **31.3** | **(±19.1)** | **7.32E-04** | **4.39E-02*^‡^*** | ***** |
| ALT (U/L) | **36.6** | **(±18.5)** | **32.4** | **(±15.3)** | **41.4** | **(±20.7)** | **5.70E-05** | **3.42E-03***^†^* | ****** |
| GGT (U/L) | 34.4 | (±21.2) | 32.2 | (±19.2) | 36.9 | (±23.0) | **3.27E-02** | 1.00*^†^* | NS |
| Total bilirubin (mg/dL) | 0.51 | (±0.22) | 0.48 | (±0.21) | 0.54 | (±0.24) | **4.00E-02** | 1.00*^†^* | NS |
| CRP (mg/dL) | 0.9 | (±0.9) | 1.0 | (±1.1) | 0.8 | (±0.7) | 0.585 | 1.00*^†^* | NS |
| CDT | 1.89 | (±0.42) | 1.93 | (±0.48) | 1.85 | (±0.33) | 0.129 | 1.00*^†^* | NS |
| *Sphingoid bases* (μM) |  |  |  |  |  |  |  |  | NS |
| C16SO | 17.884 | (±5.059) | 18.005 | (±4.912) | 17.744 | (±5.244) | 0.626 | 1.00*^†^* | NS |
| C16SA | 0.674 | (±0.278) | 0.634 | (±0.221) | 0.721 | (±0.326) | **2.93E-02** | 1.00*^†^* | NS |
| C17SO | 8.000 | (±1.874) | 8.049 | (±1.866) | 7.943 | (±1.890) | 0.658 | 1.00*^†^* | NS |
| C17SA | 0.103 | (±0.038) | 0.096 | (±0.033) | 0.111 | (±0.042) | **6.18E-03** | 0.371*^†^* | NS |
| C18SAdiene | 36.649 | (±7.923) | 37.168 | (±7.737) | 36.053 | (±8.129) | 0.243 | 1.00*^†^* | NS |
| C18SO | 76.246 | (±13.346) | 77.031 | (±13.612) | 75.344 | (±13.043) | 0.342 | 1.00*^†^* | NS |
| C18SA | 3.786 | (±1.166) | 3.701 | (±1.157) | 3.883 | (±1.174) | 0.235 | 1.00*^†^* | NS |
| C19SO | 0.235 | (±0.079) | 0.229 | (±0.078) | 0.242 | (±0.080) | 0.207 | 1.00*^†^* | NS |
| C19SA | 0.010 | (±0.006) | 0.010 | (±0.006) | 0.010 | (±0.006) | 0.909 | 1.00*^†^* | NS |
| MeC18SO | 2.522 | (±0.877) | 2.507 | (±0.804) | 2.540 | (±0.958) | 0.974 | 1.00*^†^* | NS |
| MeC18SA | 0.112 | (±0.050) | 0.104 | (±0.042) | 0.121 | (±0.056) | **2.22E-02** | 1.00*^†^* | NS |
| C18PhytoSO | 0.874 | (±0.337) | 0.892 | (±0.353) | 0.853 | (±0.318) | 0.378 | 1.00*^†^* | NS |
| C20SO | 0.180 | (±0.063) | 0.181 | (±0.062) | 0.178 | (±0.064) | 0.733 | 1.00*^†^* | NS |
| C20SA | 0.021 | (±0.015) | 0.021 | (±0.013) | 0.021 | (±0.016) | 0.903 | 1.00*^†^* | NS |
| 1-deoxySO | **0.221** | **(±0.129)** | **0.192** | **(±0.108)** | **0.254** | **(±0.143)** | **1.62E-04** | **9.71E-03***^†^* | ****** |
| 1-deoxySA | 0.133 | (±0.069) | 0.122 | (±0.060) | 0.145 | (±0.076) | **1.10E-02** | 0.662*^†^* | NS |
| C16SO-1-phosphate | 0.034 | (±0.017) | 0.035 | (±0.017) | 0.033 | (±0.018) | 0.196 | 1.00*^†^* | NS |
| C17SO-1-phosphate | 0.019 | (±0.010) | 0.019 | (±0.010) | 0.018 | (±0.009) | 0.264 | 1.00*^†^* | NS |
| C18SAdiene-1-phosphate | 0.388 | (±0.141) | 0.397 | (±0.138) | 0.378 | (±0.145) | 0.237 | 1.00† | NS |
| C18SO-1-phosphate | 0.576 | (±0.124) | 0.589 | (±0.124) | 0.562 | (±0.122) | 0.111 | 1.00*^†^* | NS |
| C18SA-1-phosphate | 0.115 | (±0.044) | 0.117 | (±0.041) | 0.113 | (±0.048) | 0.398 | 1.00*^†^* | NS |
| MeC18SO-1-phosphate | 0.010 | (±0.006) | 0.010 | (±0.006) | 0.010 | (±0.007) | 0.666 | 1.00*^†^* | NS |
| C19SO-1-phosphate | 0.004 | (±0.004) | 0.004 | (±0.003) | 0.004 | (±0.004) | 0.392 | 1.00*^†^* | NS |
| *Amino Acids (mM)* |  |  |  |  |  |  |  |  |  |
| Glycine | 0.081 | (±0.031) | 0.084 | (±0.037) | 0.077 | (±0.023) | 0.131 | 1.00*^‡^* | NS |
| Alanine | 0.143 | (±0.034) | 0.136 | (±0.033) | 0.151 | (±0.035) | **1.13E-03** | 6.77E-02*^†^* | NS |
| Serine | 0.035 | (±0.008) | 0.037 | (±0.009) | 0.034 | (±0.007) | **1.05E-02** | 0.633*^†^* | NS |
| Alanine/Serine-ratio | **4.166** | **(±1.081)** | **3.820** | **(±0.923)** | **4.564** | **(±1.116)** | **7.09E-08** | **4.26E-06***^†^* | ******** |
| Proline | 0.079 | (±0.023) | 0.077 | (±0.025) | 0.081 | (±0.022) | 0.134 | 1.00*^†^* | NS |
| Valine | 0.115 | (±0.023) | 0.111 | (±0.021) | 0.119 | (±0.025) | **9.73E-03** | 0.584*^†^* | NS |
| Threonine | 0.058 | (±0.015) | 0.058 | (±0.014) | 0.057 | (±0.016) | 0.394 | 1.00*^†^* | NS |
| Leucine | 0.027 | (±0.006) | 0.026 | (±0.005) | 0.028 | (±0.006) | **4.39E-02** | 1.00*^†^* | NS |
| Isoleucine | 0.066 | (±0.013) | 0.064 | (±0.012) | 0.068 | (±0.014) | 5.67E-02 | 1.00*^†^* | NS |
| Asparagine | 0.009 | (±0.002) | 0.009 | (±0.002) | 0.010 | (±0.002) | 0.367 | 1.00*^†^* | NS |
| Lysine | 0.097 | (±0.020) | 0.096 | (±0.021) | 0.098 | (±0.020) | 0.501 | 1.00*^†^* | NS |
| Glutamine | 0.083 | (±0.022) | 0.082 | (±0.022) | 0.084 | (±0.022) | 0.619 | 1.00*^†^* | NS |
| Methionine | 0.010 | (±0.002) | 0.010 | (±0.002) | 0.010 | (±0.002) | 0.711 | 1.00*^†^* | NS |
| Histidine | 0.045 | (±0.009) | 0.046 | (±0.010) | 0.044 | (±0.008) | 9.76E-02 | 1.00*^†^* | NS |
| Arginine | 0.031 | (±0.016) | 0.033 | (±0.018) | 0.030 | (±0.013) | 0.147 | 1.00*^†^* | NS |
| Tyrosine | **0.035** | **(±0.010)** | **0.033** | **(±0.009)** | **0.037** | **(±0.011)** | **5.47E-04** | **3.28E-02***^†^* | ***** |
| Cysteine | 0.004 | (±0.003) | 0.004 | (±0.003) | 0.004 | (±0.002) | 0.814 | 1.00*^†^* | NS |
| Phenylalanine | 0.049 | (±0.007) | 0.048 | (±0.007) | 0.050 | (±0.007) | **2.61E-02** | 1.00*^†^* | NS |

No histological evidence of metabolic dysfunction-associated steatohepatitis (No-MASH), metabolic dysfunction-associated steatohepatitis (MASH), body mass index (BMI), total cholesterol (Total-C), high-density lipoprotein cholesterol (HDL-C), low-density lipoprotein cholesterol (LDL-C), triglycerides (TG), glycated haemoglobin A1 (HbA1c), aspartate aminotransferase (AST), alanine aminotransferase (ALT), gamma-glutamyltransferase (GGT), C-reactive protein (CRP), carbohydrate-deficient transferrin (CDT), C16 sphingosine (C16SO), C16 sphinganine (C16SA), C17 sphingosine (C17SO) , C17 sphinganine (C17SA), C18 sphingadiene (C18SAdiene), C18 sphingosine (C18SO), C18 sphinganine (C18SA), C19 sphingosine (C19SO), C19 sphinganine (C19SA), omega-3-methylated C18 sphingosine (meC18SO), omega-3-methylated C18 sphinganine (meC18SA), C18 phyto-sphingosine (C18PhytoSO), C20 sphingosine (C20SO), C20 sphinganine (C20SA), 1-deoxysphingosine (1-deoxySO), 1-deoxysphinganine (1-deoxySA). Data are expressed as mean ±SD or percentage. *p* values: *^†^* Unpaired t test followed by Bonferroni multiple correction, *^‡^* Welch test followed by Bonferroni multiple correction, ^#^Pearson Chi-square. *P < 0.05, **P < 0.01, ***P<0.001, ****P<0.0001.

**Table S2** Characteristics in men without MASH versus MASH

| Men | Total  N=98 (100%) | | No-MASH  N=26 (26.5%) | | MASH  N=72 (73.5%) | | *p* value | Adjusted *p* value | Significance level |
| --- | --- | --- | --- | --- | --- | --- | --- | --- | --- |
| Age (years) | 44.6 | (±12.7) | 46.9 | (±11.7) | 43.7 | (±13.0) | 0.270 | 1.00*^†^* | NS |
| Weight (kg) | 122.1 | (±22.1) | 119.7 | (±18.8) | 123.0 | (±23.2) | 0.611 | 1.00*^†^* | NS |
| BMI (kg/m^2^) | 38.7 | (±6.2) | 38.4 | (±6.0) | 38.8 | (±6.3) | 0.796 | 1.00*^†^* | NS |
| Waist (cm) | 123.8 | (±13.3) | 121.8 | (±12.8) | 124.5 | (±13.5) | 0.362 | 1.00*^†^* | NS |
| Hip (cm) | 118.8 | (±10.2) | 118.4 | (±9.4) | 118.9 | (±10.5) | 0.847 | 1.00*^†^* | NS |
| Waist/Hip-Ratio | 1.0 | (±0.1) | 1.0 | (±0.1) | 1.1 | (±0.1) | 0.277 | 1.00*^†^* | NS |
| Total-C (mg/dl) | 195.7 | (±38.9) | 192.2 | (±47.4) | 197.0 | (±35.7) | 0.512 | 1.00*^†^* | NS |
| HDL-C (mg/dL) | 41.9 | (±8.3) | 41.3 | (±8.5) | 42.2 | (±8.3) | 0.622 | 1.00*^†^* | NS |
| LDL-C (mg/dL) | 121.5 | (±35.5) | 120.3 | (±43.1) | 121.9 | (±32.6) | 0.724 | 1.00*^†^* | NS |
| TG (mg/dL) | 161.5 | (±73.3) | 153.0 | (±74.7) | 164.6 | (±73.0) | 0.391 | 1.00*^†^* | NS |
| HbA1c (%) | 5.8 | (±0.7) | 5.7 | (±0.4) | 5.8 | (±0.7) | 0.505 | 1.00*^†^* | NS |
| Glucose (mg/dL) | 90.5 | (±32.6) | 86.0 | (±12.0) | 92.1 | (±37.3) | 0.379 | 1.00*^†^* | NS |
| Insulin (μU/dL) | 20.5 | (±14.8) | 18.9 | (±16.4) | 21.1 | (±14.2) | 0.385 | 1.00*^†^* | NS |
| Lipid lowering medication | 15 | (15.3%) | 5 | (19.2%) | 10 | (13.9%) | 0.517^#^ |  | NS |
| AST (U/L) | **38.2** | **(±19.5)** | **30.0** | **(±8.0)** | **41.2** | **(±21.4)** | **1.77E-03** | 0.106***^‡^*** | NS |
| ALT (U/L) | **61.5** | **(±33.2)** | **44.4** | **(±14.1)** | **67.7** | **(±35.9)** | **2.97E-05** | **1,78E-03*^‡^*** | ****** |
| GGT (U/L) | 54.6 | (±37.0) | 53.5 | (±43.3) | 55.0 | (±34.7) | 0.415 | 1.00*^†^* | NS |
| Total bilirubin (mg/dL) | 0.63 | (±0.27) | 0.58 | (±0.22) | 0.65 | (±0.29) | 0.280 | 1.00*^†^* | NS |
| CRP (mg/dL) | 0.6 | (±0.8) | 0.6 | (±0.6) | 0.6 | (±0.8) | 0.706 | 1.00*^†^* | NS |
| CDT | 1.88 | (±0.33) | 1.79 | (±0.20) | 1.92 | (±0.35) | 5.12E-02 | 1.00*^‡^* | NS |
| *Sphingoid bases* (μM) |  |  |  |  |  |  |  |  |  |
| C16SO | 15.087 | (±5.086) | 14.259 | (±4.397) | 15.385 | (±5.309) | 0.303 | 1.00*^†^* | NS |
| C16SA | 0.643 | (±0.283) | 0.561 | (±0.240) | 0.672 | (±0.293) | 5.26E-02 | 1.00*^†^* | NS |
| C17SO | 7.019 | (±1.946) | 6.780 | (±1.661) | 7.105 | (±2.042) | 0.541 | 1.00*^†^* | NS |
| C17SA | 0.100 | (±0.044) | 0.088 | (±0.040) | 0.104 | (±0.045) | 7.63E-02 | 1.00*^†^* | NS |
| C18SAdiene | 29.698 | (±6.738) | 28.677 | (±6.593) | 30.067 | (±6.798) | 0.296 | 1.00*^†^* | NS |
| C18SO | 71.851 | (±12.500) | 72.915 | (±13.846) | 71.466 | (±12.058) | 0.687 | 1.00*^†^* | NS |
| C18SA | 3.843 | (±1.243) | 3.638 | (±1.121) | 3.917 | (±1.284) | 0.360 | 1.00*^†^* | NS |
| C19SO | 0.234 | (±0.085) | 0.222 | (±0.089) | 0.239 | (±0.083) | 0.315 | 1.00*^†^* | NS |
| C19SA | 0.010 | (±0.006) | 0.008 | (±0.005) | 0.010 | (±0.006) | 7.87E-02 | 1.00*^†^* | NS |
| MeC18SO | 2.485 | (±0.978) | 2.422 | (±1.071) | 2.507 | (±0.950) | 0.527 | 1.00*^†^* | NS |
| MeC18SA | 0.114 | (±0.056) | 0.103 | (±0.067) | 0.118 | (±0.051) | 0.118 | 1.00*^†^* | NS |
| C18PhytoSO | 0.848 | (±0.317) | 0.829 | (±0.288) | 0.855 | (±0.328) | 0.789 | 1.00*^†^* | NS |
| C20SO | 0.197 | (±0.066) | 0.199 | (±0.072) | 0.196 | (±0.064) | 0.860 | 1.00*^†^* | NS |
| C20SA | 0.023 | (±0.014) | 0.020 | (±0.013) | 0.025 | (±0.014) | 0.196 | 1.00*^†^* | NS |
| 1-deoxySO | 0.269 | (±0.139) | 0.256 | (±0.119) | 0.274 | (±0.146) | 0.687 | 1.00*^†^* | NS |
| 1-deoxySA | 0.141 | (±0.070) | 0.141 | (±0.068) | 0.140 | (±0.071) | 0.895 | 1.00*^†^* | NS |
| C16SO-1-phosphate | 0.032 | (±0.018) | 0.031 | (±0.013) | 0.033 | (±0.019) | 0.637 | 1.00*^†^* | NS |
| C17SO-1-phosphate | 0.018 | (±0.014) | 0.017 | (±0.007) | 0.019 | (±0.015) | 0.630 | 1.00*^†^* | NS |
| C18SAdiene-1-phosphate | 0.367 | (±0.191) | 0.335 | (±0.123) | 0.379 | (±0.210) | 0.300 | 1.00*^†^* | NS |
| C18SO-1-phosphate | 0.587 | (±0.135) | 0.605 | (±0.152) | 0.581 | (±0.129) | 0.476 | 1.00*^†^* | NS |
| C18SA-1-phosphate | 0.114 | (±0.047) | 0.108 | (±0.039) | 0.116 | (±0.049) | 0.424 | 1.00*^†^* | NS |
| MeC18SO-1-phosphate | 0.010 | (±0.007) | 0.009 | (±0.006) | 0.010 | (±0.007) | 0.242 | 1.00*^†^* | NS |
| C19SO-1-phosphate | 0.003 | (±0.003) | 0.002 | (±0.002) | 0.003 | (±0.003) | 6.76E-02 | 1.00*^†^* | NS |
| *Amino Acids (mM)* |  |  |  |  |  |  |  |  |  |
| Glycine | 0.075 | (±0.013) | 0.071 | (±0.014) | 0.076 | (±0.013) | 7.55E-02 | 1.00*^†^* | NS |
| Alanine | **0.157** | **(±0.033)** | **0.140** | **(±0.030)** | **0.163** | **(±0.032)** | **1.31E-03** | 7.83E-02***^†^*** | NS |
| Serine | 0.037 | (±0.007) | 0.037 | (±0.008) | 0.037 | (±0.007) | 0.937 | 1.00*^†^* | NS |
| Alanine/Serine-ratio | **4.383** | **(±1.001)** | **3.887** | **(±0.696)** | **4.562** | **(±1.038)** | **3.67E-03** | 0.220***^†^*** | NS |
| Proline | 0.095 | (±0.022) | 0.092 | (±0.020) | 0.097 | (±0.023) | 0.398 | 1.00*^†^* | NS |
| Valine | 0.138 | (±0.025) | 0.133 | (±0.028) | 0.140 | (±0.023) | 0.212 | 1.00*^†^* | NS |
| Threonine | 0.059 | (±0.012) | 0.060 | (±0.013) | 0.059 | (±0.011) | 0.863 | 1.00*^†^* | NS |
| Leucine | 0.034 | (±0.006) | 0.032 | (±0.008) | 0.034 | (±0.006) | 0.141 | 1.00*^†^* | NS |
| Isoleucine | 0.083 | (±0.014) | 0.080 | (±0.017) | 0.084 | (±0.013) | 0.197 | 1.00*^†^* | NS |
| Asparagine | 0.010 | (±0.002) | 0.010 | (±0.002) | 0.010 | (±0.002) | 0.984 | 1.00*^†^* | NS |
| Lysine | 0.107 | (±0.021) | 0.105 | (±0.027) | 0.107 | (±0.018) | 0.777 | 1.00*^‡^* | NS |
| Glutamine | 0.104 | (±0.031) | 0.100 | (±0.028) | 0.106 | (±0.032) | 0.435 | 1.00*^†^* | NS |
| Methionine | 0.012 | (±0.003) | 0.012 | (±0.003) | 0.012 | (±0.003) | 0.408 | 1.00*^†^* | NS |
| Histidine | 0.048 | (±0.008) | 0.047 | (±0.010) | 0.048 | (±0.007) | 0.682 | 1.00*^†^* | NS |
| Arginine | 0.033 | (±0.015) | 0.034 | (±0.016) | 0.032 | (±0.015) | 0.555 | 1.00*^†^* | NS |
| Tyrosine | 0.042 | (±0.009) | 0.040 | (±0.011) | 0.043 | (±0.008) | 0.139 | 1.00*^†^* | NS |
| Cysteine | 0.004 | (±0.002) | 0.004 | (±0.002) | 0.004 | (±0.002) | 0.532 | 1.00*^†^* | NS |
| Phenylalanine | 0.053 | (±0.007) | 0.051 | (±0.009) | 0.054 | (±0.006) | 0.169 | 1.00*^‡^* | NS |

No histological evidence of metabolic dysfunction-associated steatohepatitis (No-MASH), metabolic dysfunction-associated steatohepatitis (MASH), body mass index (BMI), total cholesterol (Total-C), high-density lipoprotein cholesterol (HDL-C), low-density lipoprotein cholesterol (LDL-C), triglycerides (TG), glycated haemoglobin A1 (HbA1c), aspartate aminotransferase (AST), alanine aminotransferase (ALT), gamma-glutamyltransferase (GGT), C-reactive protein (CRP), carbohydrate-deficient transferrin (CDT), C16 sphingosine (C16SO), C16 sphinganine (C16SA), C17 sphingosine (C17SO) , C17 sphinganine (C17SA), C18 sphingadiene (C18SAdiene), C18 sphingosine (C18SO), C18 sphinganine (C18SA), C19 sphingosine (C19SO), C19 sphinganine (C19SA), omega-3-methylated C18 sphingosine (meC18SO), omega-3-methylated C18 sphinganine (meC18SA), C18 phyto-sphingosine (C18PhytoSO), C20 sphingosine (C20SO), C20 sphinganine (C20SA), 1-deoxysphingosine (1-deoxySO), 1-deoxysphinganine (1-deoxySA). Data are expressed as mean ±SD or percentage. *p* values: *^†^* Unpaired t test, *^‡^* Welch test, ^#^Pearson Chi-square. *P < 0.05, **P < 0.01, ***P<0.001, ****P<0.0001.

**Table S3** Clinical and laboratory data according to MAS score-based classification

|  | No-MASH  (MAS<3)  N=123 (39%) | | Borderline MASH  (MAS 3-4)  N=84 (26.7%) | | Definite MASH  (MAS≥5)  N=108 (34.3%) | | *p* value (Over three states) | Post hoc  (No-MASH vs. borderline MASH) | Post hoc  (MASH vs. borderline MASH) |
| --- | --- | --- | --- | --- | --- | --- | --- | --- | --- |
| Age (years) | 41.6 | (±12.2) | 44.5 | (±13.4) | 45.5 | (±11.8) | 5.09E-02*^†^* | 0.307 | 1.00 |
| Gender: men | **21** | **(17.1%)** | **30** | **(35.7%)** | **47** | **(43.5%)** | **4.78E-05^#^** | **2.24E-03** | 0.274 |
| Weight (kg) | 109.2 | (±17.6) | 110.9 | (±19.8) | 115.8 | (±21.8) | 5.08E-02*^†^* | 1.00 | 0.279 |
| BMI (kg/m^2^) | 39.0 | (±5.9) | 39.1 | (±5.6) | 39.7 | (±6.1) | 0.692*^†^* | 1.00 | 1.00 |
| Waist (cm) | **114.5** | **(±12.6)** | **119.3** | **(±12.9)** | **121.9** | **(±12.7)** | **4.18E-05*^†^*** | **2.06E-02** | 0.474 |
| Hip (cm) | **124.5** | **(±9.7)** | **121.3** | **(±10.0)** | **121.4** | **(±11.3)** | **3.06E-02*^†^*** | 9.20E-02 | 1.00 |
| Waist/Hip-Ratio | **0.9** | **(±0.1)** | **1.0** | **(±0.1)** | **1.0** | **(±0.1)** | **2.58E-10*^†^*** | **1.95E-05** | 0.374 |
| Total-C (mg/dl) | 203.7 | (±43.0) | 202.7 | (±39.5) | 203.1 | (±37.3) | 0.989*^†^* | 1.00 | 1.00 |
| HDL-C (mg/dL) | **53.2** | **(±15.2)** | **49.5** | **(±11.6)** | **46.1** | **(±14.0)** | **2.26E-04*^†^*** | 0.297 | 9.70E-02 |
| LDL-C (mg/dL) | 123.7 | (±37.8) | 123.6 | (±35.9) | 123.8 | (±34.5) | 0.995*^†^* | 1.00 | 1.00 |
| TG (mg/dL) | **129.3** | **(±58.4)** | **148.1** | **(±62.4)** | **166.2** | **(±75.6)** | **2.47E-04*^†^*** | 5.86E-02 | 0.473 |
| HbA1c (%) | **5.5** | **(±0.3)** | **5.7** | **(±0.6)** | **5.8** | **(±0.6)** | **7.09E-04*^‡^*** | 0.120 | 0.328 |
| Glucose (mg/dL) | **81.9** | **(±9.5)** | **87.0** | **(±34.7)** | **88.8** | **(±16.6)** | **9.37E-03*^†^*** | 0.297 | 0.739 |
| Insulin (μU/dL) | **14.8** | **(±10.9)** | **17.0** | **(±10.9)** | **20.9** | **(±11.9)** | **1.83E-05*^†^*** | 0.186 | **4.07E-02** |
| Medication for diabetes | 0 | (0%) | 0 | (0%) | 0 | (0%) |  |  |  |
| Lipid lowering medication | 10 | (8.1%) | 7 | (8,3%) | 17 | (15.7%) | 0.124 | 0.958^#^ | 0.124^#^ |
| AST (U/L) | **25.5** | **(±8.0)** | **28.5** | **(±13.0)** | **38.8** | **(±23.2)** | **2.76E-08*^‡^*** | 0.593 | **1.63E-04** |
| ALT (U/L) | **34.4** | **(±16.0)** | **40.2** | **(±19.2)** | **59.0** | **(±34.0)** | **2.74E-14*^‡^*** | 7.50E-02 | **5.28E-07** |
| GGT (U/L) | **36.5** | **(±27.9)** | **37.9** | **(±24.4)** | **47.5** | **(±31.3)** | **2.74E-04*^†^*** | 0.928 | **2.50E-02** |
| Total bilirubin (mg/dL) | **0.50** | **(±0.21)** | **0.56** | **(±0.27)** | **0.59** | **(±0.26)** | **2.36E-02*^†^*** | 0.258 | 1.00 |
| CRP (mg/dL) | 0.9 | (±1.0) | 0.8 | (±1.0) | 0.7 | (±0.7) | 0.302*^†^* | 1.00 | 1.00 |
| CDT | 1.90 | (±0.46) | 1.88 | (±0.33) | 1.88 | (±0.34) | 0.918*^†^* | 1.00 | 1.00 |
| *Sphingoid bases* (μM) |  |  |  |  |  |  |  |  |  |
| C16SO | 17.522 | (±5.119) | 17.239 | (±5.231) | 16.258 | (±5.295) | 0.134*^†^* | 1.00 | 0.506 |
| C16SA | **0.629** | **(±0.232)** | **0.628** | **(±0.283)** | **0.734** | **(±0.313)** | **3.96E-03*^†^*** | 1.00 | **1.51E-02** |
| C17SO | 7.926 | (±1.933) | 7.733 | (±2.027) | 7.402 | (±1.879) | 0.112*^†^* | 1.00 | 0.781 |
| C17SA | **0.097** | **(±0.035)** | **0.094** | **(±0.034)** | **0.114** | **(±0.046)** | **1.16E-03*^‡^*** | 0.872 | **2.16E-03** |
| C18SAdiene | **36.077** | **(±8.338)** | **34.864** | **(±8.973)** | **32.382** | **(±7.011)** | **2.84E-03*^†^*** | 0.711 | 0.151 |
| C18SO | 76.858 | (±14.074) | 75.000 | (±13,292) | 72.529 | (±11.850) | 5.87E-02*^†^* | 1.00 | 0.663 |
| C18SA | **3.697** | **(±1.138)** | **3.633** | **(±1.130)** | **4.057** | **(±1.258)** | **2.90E-02*^†^*** | 1.00 | 5.51E-02 |
| C19SO | 0.230 | (±0.080) | 0.234 | (±0,079) | 0.242 | (±0.083) | 0.527*^†^* | 1.00 | 1.00 |
| C19SA | 0.009 | (±0.006) | 0.009 | (±0,005) | 0.010 | (±0.007) | 0.312*^†^* | 1.00 | 0.562 |
| MeC18SO | 2.528 | (±0.859) | 2.459 | (±0,999) | 2.531 | (±0.897) | 0.659*^†^* | 1.00 | 1.00 |
| MeC18SA | **0.106** | **(±0.049)** | **0.104** | **(±0.047)** | **0.127** | **(±0.056)** | **2.01E-03*^†^*** | 1.00 | **8.45E-03** |
| C18PhytoSO | 0.886 | (±0.346) | 0.875 | (±0,326) | 0.836 | (±0.317) | 0.466*^†^* | 1.00 | 1.00 |
| C20SO | 0.184 | (±0.063) | 0.180 | (±0,059) | 0.190 | (±0.069) | 0.618*^†^* | 1.00 | 0.988 |
| C20SA | 0.020 | (±0.013) | 0.021 | (±0,012) | 0.024 | (±0.017) | 0.133*^†^* | 1.00 | 0.319 |
| 1-deoxySO | **0.199** | **(±0.111)** | **0.250** | **(±0.111)** | **0.268** | **(±0.162)** | **1.22E-04*^†^*** | **4.66E-03** | 1.00 |
| 1-deoxySA | **0.123** | **(±0.060)** | **0.139** | **(±0.056)** | **0.147** | **(±0.085)** | **2.02E-02*^‡^*** | **4.55E-02** | 0.970 |
| C16SO-1-phosphate | 0.034 | (±0.016) | 0.033 | (±0,018) | 0.033 | (±0.018) | 0.489*^†^* | 1.00 | 1.00 |
| C17SO-1-phosphate | 0.019 | (±0.010) | 0.018 | (±0,009) | 0.018 | (±0.013) | 0.481*^†^* | 1.00 | 1.00 |
| C18SAdiene-1-phosphate | 0.391 | (±0.138) | 0.374 | (±0,144) | 0.377 | (±0.189) | 0.531*^†^* | 0.835 | 1.00 |
| C18SO-1-phosphate | 0.589 | (±0.128) | 0.584 | (±0,138) | 0.566 | (±0.118) | 0.397*^†^* | 1.00 | 1.00 |
| C18SA-1-phosphate | 0.116 | (±0.041) | 0.119 | (±0,046) | 0.111 | (±0.048) | 0.374*^†^* | 1.00 | 1.00 |
| MeC18SO-1-phosphate | 0.009 | (±0.006) | 0.011 | (±0,006) | 0.010 | (±0.007) | 0.421*^†^* | 1.00 | 0.790 |
| C19SO-1-phosphate | 0.003 | (±0.003) | 0.004 | (±0,004) | 0.004 | (±0.003) | 0.564*^†^* | 0.577 | 1.00 |
| *Amino Acids (mM)* |  |  |  |  |  |  |  |  |  |
| Glycine | 0.083 | (±0.035) | 0.076 | (±0.022) | 0.076 | (±0.019) | 0.269*^‡^* | 0.464 | 1.00 |
| Alanine | **0.135** | **(±0.032)** | **0.149** | **(±0.029)** | **0.160** | **(±0.036)** | **6.19E-08*^†^*** | **3.75E-03** | 9.72E-02 |
| Serine | 0.037 | (±0.008) | 0.035 | (±0.007) | 0.035 | (±0.007) | 0.124*^†^* | 0.164 | 1.00 |
| Alanine/Serine-ratio | **3.750** | **(±0.838)** | **4.404** | **(±0.917)** | **4.653** | **(±1.172)** | **1.29E-11*^†^*** | **1.85E-06** | 0.477 |
| Proline | **0.078** | **(±0.023)** | **0.084** | **(±0.025)** | **0.090** | **(±0.024)** | **6.01E-04*^†^*** | 0.147 | 0.335 |
| Valine | **0.113** | **(±0.025)** | **0.120** | **(±0.022)** | **0.133** | **(±0.027)** | **6.30E-08*^†^*** | 0.109 | **3.29E-03** |
| Threonine | 0.058 | (±0.014) | 0.056 | (±0.011) | 0.059 | (±0.015) | 0.481*^†^* | 1.00 | 0.722 |
| Leucine | **0.027** | **(±0.006)** | **0.029** | **(±0.006)** | **0.032** | **(±0.007)** | **2.39E-07*^†^*** | 8.82E-02 | **8.92E-03** |
| Isoleucine | **0.066** | **(±0.015)** | **0.071** | **(±0.014)** | **0.077** | **(±0.016)** | **1.39E-06*^†^*** | **4.39E-02** | **4.81E-02** |
| Asparagine | 0.009 | (±0.002) | 0.010 | (±0.002) | 0.010 | (±0.003) | 6.58E-02*^‡^* | 0.700 | 0.827 |
| Lysine | 0.097 | (±0.023) | 0.100 | (±0.017) | 0.104 | (±0.021) | 6.21E-02*^‡^* | 0.741 | 0.773 |
| Glutamine | **0.085** | **(±0.024)** | **0.086** | **(±0.023)** | **0.097** | **(±0.031)** | **1.07E-03*^†^*** | 1.00 | **1.80E-02** |
| Methionine | **0.010** | **(±0.002)** | **0.011** | **(±0.002)** | **0.011** | **(±0.003)** | **3.02E-03*^†^*** | 0.499 | 0.236 |
| Histidine | 0.046 | (±0.010) | 0.046 | (±0.007) | 0.046 | (±0.008) | 0.981*^†^* | 1.00 | 1.00 |
| Arginine | 0.033 | (±0.018) | 0.030 | (±0.013) | 0.032 | (±0.015) | 0.449*^†^* | 0.620 | 1.00 |
| Tyrosine | **0.034** | **(±0.009)** | **0.038** | **(±0.008)** | **0.041** | **(±0.011)** | **1.77E-08*^†^*** | **3.23E-03** | 6.20E-02 |
| Cysteine | 0.004 | (±0.003) | 0.004 | (±0.002) | 0.004 | (±0.002) | 0.108*^†^* | 1.00 | 0.133 |
| Phenylalanine | **0.048** | **(±0.007)** | **0.051** | **(±0.006)** | **0.052** | **(±0.007)** | **1.75E-05*^†^*** | **1.72E-02** | 0.369 |

No histological evidence of metabolic dysfunction-associated steatohepatitis (No-MASH), borderline steatohepatitis (borderline MASH), steatohepatitis (MASH), MASLD activity score (MAS), body mass index (BMI), total cholesterol (Total-C), high-density lipoprotein cholesterol (HDL-C), low-density lipoprotein cholesterol (LDL-C), triglycerides (TG), glycated haemoglobin A1 (HbA1c), aspartate aminotransferase (AST), alanine aminotransferase (ALT), gamma-glutamyltransferase (GGT), C-reactive protein (CRP), carbohydrate-deficient transferrin (CDT), C16 sphingosine (C16SO), C16 sphinganine (C16SA), C17 sphingosine (C17SO) , C17 sphinganine (C17SA), C18 sphingadiene (C18SAdiene), C18 sphingosine (C18SO), C18 sphinganine (C18SA), C19 sphingosine (C19SO), C19 sphinganine (C19SA), omega-3-methylated C18 sphingosine (meC18SO), omega-3-methylated C18 sphinganine (meC18SA), C18 phyto-sphingosine (C18PhytoSO), C20 sphingosine (C20SO), C20 sphinganine (C20SA), 1-deoxysphingosine (1-deoxySO), 1-deoxysphinganine (1-deoxySA). Data are expressed as mean ±SD or percentage. *p* values: *^†^* ANOVA (Bonferroni corrected post hoc tests), *^‡^* Welch-ANOVA (Games-Howell corrected post hoc tests), ^#^Pearson Chi-square.
